# Supplementary material for: Viral DNA Accumulation Regulates Replication Efficiency of Chlorovirus OSy-NE5 in Two Closely Related Chlorella variabilis Strains
Source: Viruses. 2023 Jun 9;15(6):1341. doi: 10.3390/v15061341 (PMC10302427; doi:10.3390/v15061341)
Supplement: Supplementary file 1 [file viruses-15-01341-s001.zip › viruses-2434235-supplementary.pdf]

viruses

MDPI

Supplementary Materials

Article

Viral DNA Accumulation Regulates Replication Efficiency of *Chlorovirus* OSy-NE5 in Two Closely Related *Chlorella variabilis* strains

Ahmed Esmael[1,2], Irina V. Agarkova[1,3], David D. Dunigan[1,3], You Zhou[4], and James L. Van Etten[1,3,*]

[1] Nebraska Center for Virology, University of Nebraska, Lincoln, NE 68583, USA;
[2] Botany and Microbiology Department, Faculty of Science, Benha University, Benha, 13518, Egypt.
[3] Department of Plant Pathology, University of Nebraska, Lincoln, NE 68583, USA.
[4] Center for Biotechnology, University of Nebraska-Lincoln, Lincoln, NE 68588, USA.
* Correspondence: jvanetten1@unl.edu, Tel: (+1) 402-540-3474

Table S1. Primer sequences of two genes in OSy-NE5 virus and their homologs in PBCV-1.

Virus	Gene	Sequence (5′->3′)	Expected Amplicon size (nt)
OSy-NE5	OS5_104L	**Fwd**: ACTCAACCTCAACATGCTCG<br>**Rev**: TTGGGATTCTCAAACCTCAGG	135
	OS5_154L	**Fwd**: AGGAATTTCTGGGCTACTGC<br>**Rev**: GGTATTACTTTCCGTGCCCTG	146
PBCV-1	A208R	**Fwd**: GCATTTGGCGTTCTCAACC<br>**Rev**: GTGCTCGTCATCGGTATCTTC	123
	A312L	**Fwd:** AGTTCACGAGACTTCTGCTTG<br>**Rev:**<br>CCTGTCTTTCCGTGGTTATGAG	138

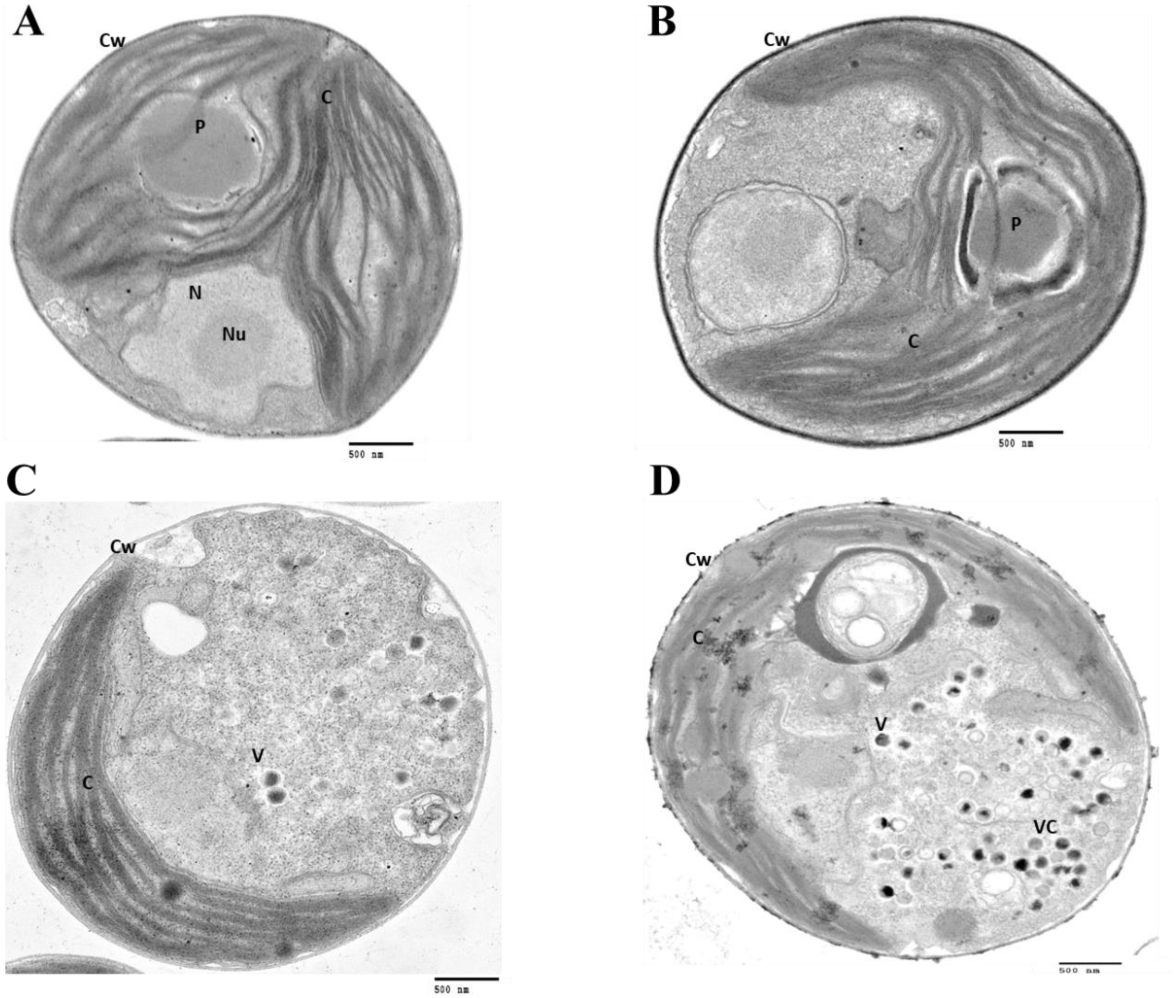

Figure S1. Transmission electron micrographs of PBCV-1 infected and uninfected *C. variabilis* Syngen 2-3. **(A)** uninfected Syngen 2-3 cell, **(B-D)** 1 h, 3 h and 6 respectively after infection with PBCV1. Symbols: chloroplast, C, pyrenoid, P, nucleus, N, nucleolus, Nu, Cell wall, Cw, virus assembly center, VC and Virus particles, V. Scale bars, 500 nm.

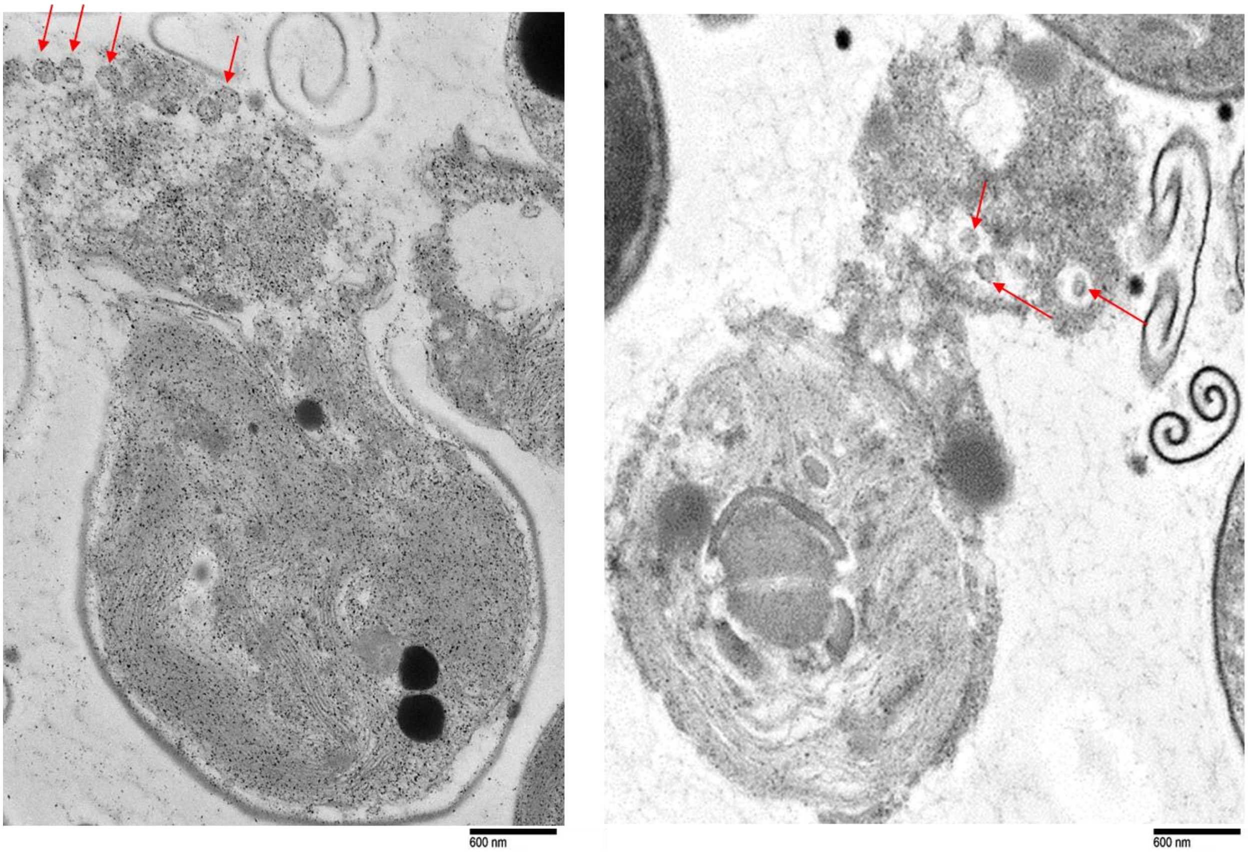

Figure S2. Transmission electron micrographs of *C. variabilis* NC64A cells infected with OSy-NE5 at 24 h p.i. The micrograph shows two different cells bursting empty OSy-NE5 capsids as indicated by the red arrows. Scale bars, 600 nm.
